# Femoral brucellosis mimicking a tumor

**DOI:** 10.1111/1759-7714.14416

**Published:** 2022-04-09

**Authors:** Yuhui Liu, Shuanghu Yuan, Yong Huang

**Affiliations:** ^1^ Department of Radiology Shandong Cancer Hospital and Institute, Shandong First Medical University and Shandong Academy of Medical Sciences Jinan Shandong People's Republic of China; ^2^ Department of Radiation Oncology Shandong Cancer Hospital and Institute, Shandong First Medical University and Shandong Academy of Medical Sciences Jinan Shandong People's Republic of China

A 63‐year‐old male farmer experienced right leg pain for 1 year. The pain initially only occurred during activity, but developed into persistent sharp pain. After 1 month of worsening pain, he was admitted to our hospital. On physical examination, the distal right femur was tender, with no warmth or erythema. Routine blood examination results were normal. Radiography (Figure 1a) and computed tomography (Figure 1b) showed expansive bone destruction with surrounding osteosclerosis in the distal right femur. Magnetic resonance imaging showed that the edge of the lesion was “map‐like” (Figure 1c). A multidisciplinary team suspected a benign femoral tumor and proposed surgical treatment. However, a radiologist questioned the diagnosis when the patient mentioned that he raised sheep. Further inquiry revealed that the patient kept cattle and sheep and had been diagnosed with brucellosis 7 years previously at a local infectious disease hospital. After antibiotic therapy, he had been considered cured and was intermittently treated with tetracycline. Computed tomography (CT) (Figure 1d) showed old bone destruction of the thoracic vertebrae. He had deliberately hidden his history of brucellosis when he was admitted to our hospital for fear of isolation for a suspected infectious disease. After learning this important personal history, we performed a femoral puncture biopsy. Pathological examination of the femoral puncture biopsy revealed dead bone. A diagnosis of brucellosis was confirmed by a serum agglutination test. After 2 months of antibiotics and hormone therapy, the patient's symptoms were relieved. People with brucellosis usually have obvious infectious symptoms, such as fever, fatigue, and leukocytosis. Our patient had no infectious symptoms at the time of presentation, which may be related to his intermittent tetracycline use. Brucellosis of the femur is easily misdiagnosed as a bone tumor. A detailed personal history is essential to ensure a correct diagnosis.

**FIGURE 1:**
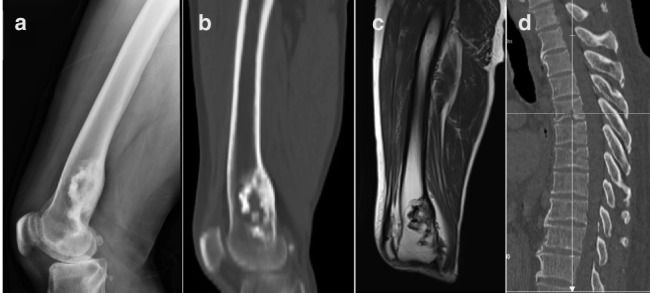
A 63‐year‐old male farmer presented with a history of gradually progressive right leg pain which he had experienced for 1 year. The pain initially only occurred during activity, but developed into persistent sharp pain. After 1 month of worsening pain, he was admitted to our hospital. On physical examination, the distal right femur was tender, with no warmth or erythema. Routine blood examination results were normal. (a) Radiography and (b) computed tomography showed expansive bone destruction with surrounding osteosclerosis in the distal right femur. (c) Magnetic resonance imaging showed that the edge of the lesion was “map‐like.” (d) Computed tomography showed old bone destruction of the thoracic vertebrae

## CONSENT

The patient provided written informed consent for the publication of this case.

## CONFLICT OF INTEREST

We declare no competing interests.

